# Current antiviral agents against human adenoviruses associated with respiratory infections

**DOI:** 10.3389/fped.2024.1456250

**Published:** 2024-08-29

**Authors:** Lexi Li, Zhengde Xie, Lili Xu

**Affiliations:** ^1^ Beijing Key Laboratory of Pediatric Respiratory Infection Diseases, Key Laboratory of Major Diseases in Children, Ministry of Education, National Clinical Research Center for Respiratory Diseases, National Key Discipline of Pediatrics (Capital Medical University), Beijing Pediatric Research Institute, Beijing Children's Hospital, Capital Medical University, National Center for Children's Health, Beijing, China; ^2^ Research Unit of Critical Infection in Children, Chinese Academy of Medical Sciences, Beijing, China

**Keywords:** human adenovirus (HAdV), antiviral agents, respiratory infection, therapies, nucleoside (acid) analogues

## Abstract

Human adenoviruses (HAdVs) are important pathogens responsible for respiratory infections. In children and immunocompromised patients, respiratory infections can cause considerable morbidity and mortality. Currently, there are no approved effective and safe antiviral therapeutics for the clinical treatment of HAdV infections, even those that have undergone preclinical/clinical trials. However, many compounds and molecules with anti-HAdV activity have been explored, and some candidates are undergoing clinical development. Here, we reviewed the reported *in vitro* and *in vivo* efficacies, as well as the therapeutic potential of these antiviral compounds, providing an overview and a summary of the current status of anti-HAdV drug development.

## Introduction

Human adenovirus (HAdV), a member of the family *Adenoviridae*, plays an important role in paediatric respiratory tract infections, especially severe pneumonia, accounting for 3.5%–11% of childhood community-acquired pneumonia (CAP) cases (1). HAdV infections can be severe or even fatal in both immunocompetent and immunocompromised patients (2). Moreover, 14%−60% of paediatric patients with HAdV pneumonia have varying degrees of pulmonary sequelae (3). To date, 116 HAdV genotypes have been identified and classified into seven species (A-G) based on immunological and genomic criteria (4). Different HAdVs have different tissue tropisms that are associated with different clinical manifestations (2, 5). HAdV species B (HAdV-3, 7, 11, 14, 16, 21, 50, 55), C (HAdV-1, 2, 5, 6) and E (HAdV-4) are mainly related to respiratory diseases (6). HAdV-3 and -7 are the most frequent HAdV causing respiratory tract infection in China, but there are obvious differences in various regions (7–9).

Despite the medical threat posed by HAdV, currently available antiviral agents and immune therapeutic approaches have not been able to successfully combat the life-threatening course of invasive HAdV infections in the immunocompromised clinical setting. Over the last decade, numerous efforts have been made to develop effective antivirals targeting HAdV. In this review, we outline HAdV biology and focus on current developments in the field of antiviral drugs for treating respiratory infections associated with HAdV.

## Virology

**Figure 1:**
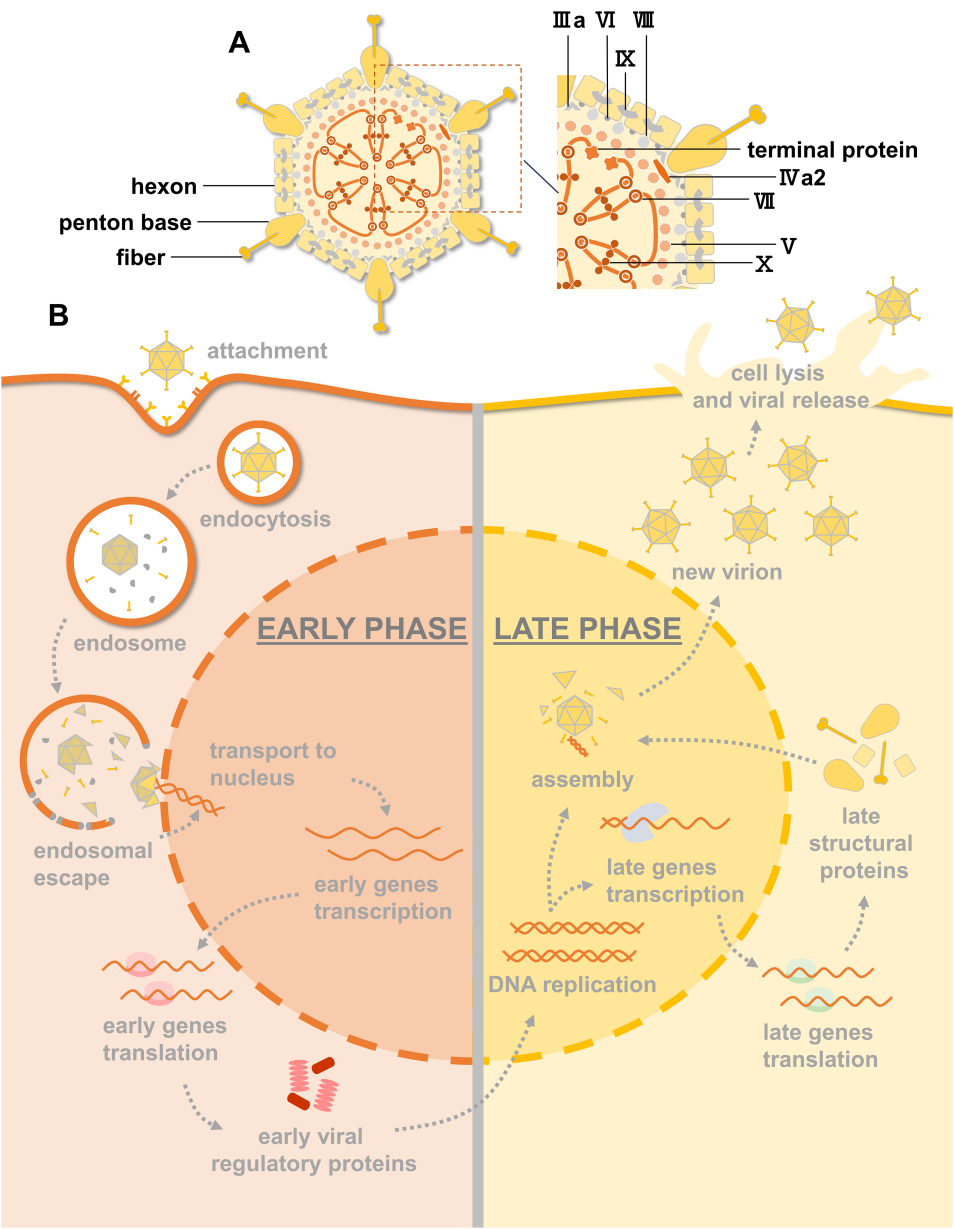
HAdV structure, infection and replication pathway. **(A)** An illustration of the HAdV particle showing the principal capsid proteins. **(B)** Overview of the HAdV replication cycle. Early and late phases of the cycle are indicated. Viral DNA replication marks the progression from early to late transition.

HAdV, which is approximately 90 nm in size, is a nonenveloped virus with a characteristic icosahedral morphology. Its structure consists of three major proteins (Figure 1A). Hexon is the most abundant coat protein, making up the triangular facets of the capsid, with a penton base at every vertex and a thin fibre projecting from each penton base. Other minor components, such as IIIa, VI, VIII and IX, mainly act as capsid cement. Inside the capsid are virion proteases, which play a vital role in the assembly of the virion, and the double-stranded DNA (dsDNA) genome is associated with five polypeptides [terminal protein (TP), V, VII, IVa2, and X] (10, 11).

Infection by HAdV, depicted in Figure 1B, starts with the binding of fibers to primary receptors on the cell surface. These receptors include coxsackie-adenovirus receptor (CAR), CD46, and desmohlein-2 (DSG-2) (12). The penton base interacts with cellular integrins to stimulate endocytosis. To avoid endosomal degradation, fibres are removed by disruption of primary receptor interactions, exposing protein VI, which mediates endosomal escape into the cytoplasm. After escaping endosomal degradation, the virion migrates to the nucleus, and the capsid proteins are disassembled during transport such that only the condensed viral genome enters the nucleus through the nuclear pore.

The HAdV replication cycle can be divided into early and late phases according to the times at which particular viral genes are expressed (10). Shortly after infection, early gene transcription units are activated and give rise to early proteins that activate other viral genes and alter the expression of host proteins for DNA synthesis. After the onset of DNA replication, activation of the HAdV major late promoter (MLP) mediates the transcription of late genes that encode viral structural proteins and proteins that are required for the maturation of virus particles. Virions are assembled in the nucleus of infected cells, and HAdV progeny are released by cell lysis (13).

## Current therapies

Treatment options are usually symptomatic, with the administration of general treatments, respiratory support, and a reduction in immunosuppression when relevant (14). Whether to supplement treatment regimens with antiviral drugs remains controversial, but the European Respiratory Society recommends antiviral drugs for critically ill patients (15). Thus, several off-label DNA/RNA synthesis-inhibiting antivirals are currently used off-label to treat severe HAdV infections in the clinic. In Table 1, a literature survery was given of reported anti-HAdV agents, with their respective anti-HAdV activities.

**Table 1:** Anti-HAdV compounds and molecules discussed in this review.

Category	Compound	HAdV genotypes related with respiratory infections	EC_50_(µM)^a^	CC_50_(µM)^a^	Reference(s)
Nucleoside/nucleotide analogues	Cidofovir	1–7,11,14	17–81	1,000	(17)
		3,5,7	0.5–1.3	>317^b^	(18)
		2	2–3.1	83	(19)
		5	4	97	(20)
		4, 5, 6, 7	1.9–28.46		(21)
		7,55	5.1–6.1	97.5	(22)
		5	34	2,264	(23)
		5	24.1	50.6	(24)
	Brincidofovir	3,5,7	<0.009–0.02	39	(18)
	Ribavirin	1,2,5,6	48–108	400	(17)
		2	>250	30	(19)
	Ganciclovir	2	35–39	115	(19)
	Zalcitabine	2	1.4–3.3	231	(19)
		2, 4	3.95–4.29	381.9	(54)
		2	<0.03	0.83	(20)
		3,11	35.5–67.2	1,227.2	(55)
		3,11	126.4–133.5	1,278.3	(56)
	Alovudine	2	0.71–3.2	231	(19)
	Filociclovir	5	66	>100	(20)
		4, 5, 6, 7	1.24–3.6	>100–150	(21)
	USC-087	3,5,6,7	0.002–0.025	1	(58)
	Stavudine	5	12.3		(59)
	6-azacytidine	5	2	766	(60)
	Gemcitabine	5			(61)
	Decitabine	5			(61)
	Cytarabine	4, 5, 7			(62)
Natural compounds	Cardamomin	3, 5	2.4–4	>90	(65)
	Nuciferine	5	4.5	>90	(65)
	Shikonin	3		28.91	(66)
	Caffeic acid	3,11	78.8–>1,110.1	57 132.5	(67)
	*p*-coumaric acid	3,11	266.8–>1,218.3	2,978.8	(67)
	Chlorogenic acid	3, 11	37.5–214.5	11 275.4	(67)
	Ferulic acid	11	120.0	476.9	(67)
	Phenolic compound extracts of *Camellia sinensis* Kuntze	5	6.62^c^	165.95^c^	(68)
	N-butanol fraction	5	2.16^c^	264.7^c^	(69)
	Gallic acid	5	27.5	290.0	(69)
	Methanol and methanol/H_2_O	5			(70)
	Dioscin	5			(71)
	Camptothecin	5			(72)
	Quercetin	3, 11	80.4–148.2	1,644.1	(55)
		3	111.2	1,379.7	(73)
	Apigenin	3, 11	41.1–77.3	221.7	(56)
	Linalool	3, 11	109.6–158.2	1,148.1	(56)
Epigenetic regulators inhibitors	Valproic acid	5			(74)
	Vorinostat	4, 5, 7			(75)
	Trichostatin A	4, 5, 7			(75)
	Chaetocin	4, 5, 7			(61)
	Lestaurtinib	4, 5, 7			(61)
	OG-L002	5			(76)
	GSK126/GSK343	5			(77)
Steroid-based compounds	Digoxin	4, 5, 7	0.02		(62)
		5	0.077		(78)
	Digitoxin	4, 5, 7	0.064		(62)
	Lanatoside C	4, 5, 7	0.032		(62)
	Dexamethasone	5			(62)
	Flunisolide	5			(62)
	Mifepristone	5, 16	1.9	270.2	(80)
	Dehydroepiandrosterone	5	88	5,530	(23)
Others	Piperazine derivatives	5	0.6–5.1	210.4	(24)
		5,16	0.8–1.3	194	(81)
		5,16	1.1–4.8	130.8–199.8	(82)
	NMSO3	2, 4, 8, 37	0.40–0.71	>1,000	(54)
	Water-soluble polymer complex of arbidol	3		435–480^c^	(83)
	Niclosamide	5, 16	0.45–0.60	3.30	(84)
	Rafoxanide	5, 16	1.30–1.38	48.89	(84)
	Ivermectin	3	9.82		(85)
	Tanespimycin (17-AAG)	3	0.5	>5	(86)
	Flavopiridol	2	0.014	0.15	(89)
	Olomoucine Ⅱ	4	2.4	>100	(90)
	Indirubin-3’-monoxime	7,55	2.2–2.0	169.1	(22)
	FIT-039	5			(91)
	LDC4,297	2	0.25	5.69	(92)
	Mycophenolic acid	5	0.05	175	(93)
	A3	5		268	(94)
	Tazarotene	5, 7, 55	8.34–13.75	98.66	(95)
	Verdinexor (KPT-335)	3,5	0.15–0.61	1.58	(86)
		5	0.03	0.1	(96)
	[Co(NH_3_)_6_]Cl_3_	7			(97)
	Nelfinavir	2, 14	0.37	25.7	(98)

^a^ EC_50_: 50% effectiveness concentration; CC_50_: 50% cytotoxicity concentration.

^b^ Maximum concentration of compound to test did not find the corresponding value.

^c^ Concentration of compound in µg/ml.

Cidofovir (CDV), an approved antiviral agent against human cytomegalovirus (HCMV), was a nucleotide analogue of cytosine that selectively inhibited viral DNA polymerase and subsequent viral replication by competitively incorporating its active metabolite into the viral DNA chain (16, 17). Several studies have proven the antiviral activity of CDV by using various *in vitro* methodologies and different host cell lines. In general, these reported 50% effectiveness concentration (EC_50_) values of CDV against different HAdV genotypes fall in the range of 0.5–81 µM (17–24). Moreover, several case reports and larger retrospective studies have presented partially effective but promising results among immunocompromised adults and paediatric patients (25–33). The success rates are highest when antiviral treatment is initiated rapidly after the diagnosis of HAdV infection (34–36). However, its low bioavailability and nephrotoxicity remain limiting factors for its widespread use. Although renal toxicity is counteracted by the concomitant use of intravenous immunoglobulin (IVIG) and renal protective measures, HAdV disease manifestations are particularly severe in children and immunocompromised individuals, who are also more susceptible to nephrotoxicity.

Current efforts are concentrated on optimizing the formulation of CDV and the development of nucleoside analogue derivatives. Brincidofovir (BCV, previously named CMX001) is a phospholipid conjugate of CDV with improved oral delivery, increased cellular uptake and reduced kidney accumulation. It has been proven to be more effective against HAdV-3/5/7 in cell culture experiments (18). Toth et al. demonstrated that BCV repressed HAdV-5 replication in the liver, salivary gland, and pancreas of immunosuppressed hamsters and suppressed HAdV-induced morbidity and mortality (37). Due to its *in vitro* and *in vivo* efficacy, it received the Fast Track designation for the treatment of HAdV infections. Subsequently, the results from phase Ⅰ trials illustrated its oral bioavailability and safety profile, while phase Ⅱ trials revealed no statistically significant difference from the placebo treatment (38, 39). Notably, BCV-related gastrointestinal toxicity has also been reported (40).

Other drugs, such as ribavirin and ganciclovir, are also nucleotide analogues but have failed to show consistent activity against HAdV (41). Specifically, ribavirin was shown to have antiviral effects against HAdV-5 *in vitro*, while it has also been reported to be largely ineffective in immunosuppressed Syrian hamsters, in which CDV and BCV are effective (17, 42). Moreover, a number of case reports mentioned successful therapy with ribavirin (43–46), but in a few small treatment studies, no clear benefit was demonstrated (47–49). These variable outcomes may be partially explained by genotype-related differences in the anti-HAdV activity of ribavirin (17, 19). For ganciclovir, a common antiviral compound against HCMV and herpes simplex virus, it has been reported that patients who received ganciclovir prophylactically or as a preemptive therapy against HCMV had lower rates of HAdV infection than other patients (50). However, ganciclovir must first undergo phosphorylation to an active form by a viral kinase, which HAdV does not encode (51). Ying et al. demonstrated that ganciclovir was effective against HAdV-5 infection both *in vitro* and in immunosuppressed hamsters, possibly because of the direct inhibition of HAdV DNA polymerase (52). However, the reported EC_50_ value was three times greater than the plasma concentration achievable with a standard dose of ganciclovir, suggesting that the *in vivo* anti-HAdV effects were rather improbable (19, 53). In conclusion, the broadly acting antivirals available in the clinic show insufficient efficacy and/or safety against HAdV infections.

## Potential antiviral agents

### Nucleoside/nucleotide analogues

Apart from CDV, BCV, ribavirin, and ganciclovir, many studies have focused on the anti-HAdV activity of other nucleotide analogues. A study performed in human embryonic lung fibroblast cells showed that several nucleotide analogues, such as zalcitabine and alovudine, were potential candidates for the treatment of HAdV-2 infections (19). Another study also confirmed the anti-HAdV-2/3/4/11 activity of zalcitabine *in vitro* (20, 54–56). In a mouse pneumonia model, zalcitabine was associated with a statistically significant reduction in the frequency of pneumonia caused by HAdV-2 infection (57). Filociclovir, a nucleotide analogue that has successfully completed phase Ⅰ clinical trials, was shown to be a potent inhibitor of HAdV-5 in human foreskin fibroblasts (HFFs) (20). Further studies have demonstrated that filociclovir is a broad-spectrum inhibitor of HAdV types 4/5/6/7 *in vitro* and is highly potent against HAdV-6 in Syrian hamsters (21). USC-087, an N-alkyl tyrosinamide phosphonate ester prodrug of HPMPA, the adenine analogue of CDV, can also protect Syrian hamsters against intravenous challenge with HAdV-5/6 (58). The anti-HIV agent stavudine and all 12 of its derivatives were found to have potent effects against HAdV-5 (59). Another HIV-effective antiviral, 6-azacytidine, appeared to inhibit HAdV-5 infection in Hep-2 cells and in Syrian hamsters (60). Additional nucleotide analogues that have anti-HAdV-4/5/7 activity, including gemcitabine, decitabine, and cytarabine, were screened from various compound libraries by using different assays (61, 62).

### Natural compounds

In recent years, a large number of natural compounds isolated from medicinal plants have been found to have potential *in vitro* and *in vivo* antiviral activities (63, 64). For example, a chemiluminescence-based, high-throughput screening (HTS) assay was developed by Wen et al., and two Chinese medicine small molecule compounds, cardamomin and nuciferine, were confirmed to have efficacious inhibitory effects on HAdV-3/5 *in vitro* (65). Cardamomin also inhibited HAdV-5 infection in BALB/c mice, indicating that Chinese herbal medicine and its natural products are rich sources of novel antiviral compounds. Similarly, the anti-HAdV-3 activity of *Radix lithospermi* was due to shikonin, which can inhibit the expression of hexon protein (66). Four phenolic compounds of *Plantago major*, caffeic acid, *p*-coumaric acid, chlorogenic acid, and ferulic acid, possessed antiviral activity against HAdV-3/11 (67). Similar to caffeic acid, treatment with phenolic compounds extracted from *Camellia sinensis* Kuntze led to a reduction in HAdV-5 replication without interfering with virus attachment (68). Furthermore, a number of other medicinal plant compounds have been shown to inhibit HAdV-5 in cell culture. These included the n-butanol fraction and gallic acid extracted from *Punica granatum* L., methanol and methanol/H_2_O extracted from *Peucedanum salinum*, dioscin extracted from air potato, and camptothecin extracted from *Camptotheca acuminata* Decne (69–72). Quercetin extracted from both *Caesalpinia pulcherrima* Swartz and *Allium* plants and apigenin and linalool extracted from *Ocimum basilicum* showed antiviral activity against HAdV-3/11 (55, 56, 73).

### Epigenetic regulators inhibitors

Cellular epigenetic modifiers involved in the transition of the HAdV genome associated with histones to a chromatin-like structure and the regulation of viral gene expression may serve as valid targets for interfering with HAdV replication. Höti et al. reported for the first time that valproic acid, a well-known class Ⅰ histone deacetylase (HDAC) inhibitor, decreased the HAdV-5 titre and inhibited viral spread (74). Other pan-HADC inhibitors, such as vorinostat and trichostatin A, were found to have inhibitory effects on HAdV-4/5/7 (75). Furthermore, Saha and Parks performed HTS to identify compounds that affect HAdV-5 function by using a library composed of described cellular epigenetic regulatory proteins (61). In addition to HDAC inhibitors, decitabine/gemcitabine (a nucleotide analogue), chaetocin (a histone methyltransferase inhibitor), and lestaurtinib (a JAK2/PRK kinase inhibitor) were also shown to significantly reduce HAdV-4/7 gene expression. Some methyltransferase inhibitors have also shown efficacy against HAdV. For instance, OG-L002, which inhibits lysine-specific demethylase 1, and GSK126/GSK343, which inhibit the EZH1/2 histone methyltransferase machinery, can inhibit the expression of the HAdV-5 early gene E1A (76, 77).

### Steroid-based compounds

In an early report, the potential of cardiotonic steroids as antiviral agents was recognized, and the investigators reasoned that ionic changes resulting from treatment with digoxin, which is also a cellular Na^+^/K^+^ ATPase inhibitor, would prevent the replication of DNA viruses, including HAdV-5 and three other herpesviruses (78). Later, digoxin and digitoxin were confirmed to alter E1A RNA splicing early to block HAdV-5 replication and reduce the viral titre (79). A fluorescence-based HTS platform was used for the identification of novel anti-HAdV compounds, and three cardiotonic steroids, digoxin, digitoxigenin and lanatoside C, were the top hits (62). These three compounds reduced viral gene expression, DNA replication, and HAdV-4/5/7 yields in A549 and MRC-5 cells. In addition to cardiotonic steroids, corticosteroids, including dexamethasone and flunisolide, were also identified as positive hits in this screen. However, subsequent validation experiments showed that neither of these compounds exhibited antiviral activity against HAdV-4/7 in MRC-5, resulting in a reduction in both the number of HAdV-5 early/late proteins and the yield of HAdV-5 in A549 cells. Mifepristone, a synthetic steroid that is structurally closely related to glucocorticoids, showed significant *in vitro* antiviral activity against HAdV-5/16 and inhibited the infection of mice with HAdV-based vectors containing a luciferase reporter gene (80). Further mechanistic assays suggested that it can affect microtubule transport, interfering with the entry of HAdV into the nucleus and therefore inhibiting HAdV infection. Natural steroid hormones, such as dehydroepiandrosterone and epiandrosterone analogues, can also exert anti-HAdV-5 activity by affecting HAdV protein synthesis and viral mature particle formation (23).

### Others

In addition to those described above, various compounds and molecules targeting every stage of the replication cycle have shown anti-HAdV activities. For example, many piperazine derivatives have anti-HAdV-5/16 activity, targeting different steps in the HAdV replication cycle, such as HAdV genome accessibility to the nucleus, early gene transcription, HAdV DNA replication, and new HAdV particle assembly, maturation and release (24, 81, 82).

#### Targeting entry

The sulfated sialic acid derivative NMSO3 inhibited HAdV-2/4, and the mechanism of its anti-HAdV activity involved the inhibition of viral absorption to cells by binding to viral particles (54).

Water-soluble complexes synthesized between arbidol and polymer compounds retained the high level of anti-HAdV-3 activity of arbidol, which could inhibit fusion of the viral lipid shell with membranes of endosomes located within the cell (83).

The salicylanilide drugs niclosamide and rafoxanide mainly block HAdV-5/16 infection at some point between endosomal escape and HAdV DNA release to the nucleus (84).

#### Targeting the early phase of infection

Ivermectin is an antiparasitic agent that has broad-spectrum antiviral activity, including activity against HAdV. Specifically, ivermectin disrupted the binding of the E1A protein to importin-α, preventing E1A import into the nucleus and therefore significantly reducing HAdV-3 replication (85).

Tanespimycin (17-AAG), an inhibitor of the HSP90 chaperone, was also reported to inhibit HAdV-5 replication, and its anti-HAdV activity might be explained by the reduction in E1A levels (86, 87).

The critical roles of cyclin-dependent kinases (CDKs), which are involved in the regulation of the cell cycle and transcription, have made them attractive targets for the development of antiviral drugs (88). Both pan-CDK inhibitors and other highly selective CDK inhibitors have been reported to suppress HAdV replication. The pan-CDK inhibitor flavopiridol blocked HAdV-2/5 infections (89). Olomoleucine Ⅱ, a derivative of another pan-CDK inhibitor, roscovitine, not only inhibits the replication of HAdV-4 independently but also almost completely eliminates HAdV-4 spread when used in combination with CDV (90). Indirubin-3’-monoxime, the derivative of a bisindole alkaloid indirubin, could inhibit various CDKs. It also exhibited potent antiviral effects against HAdV-7/55 *in vitro* and *in vivo*, and was founded to inhibit HAdV replication by downregulating RNA polymerase Ⅱ C-terminal domain phosphorylation to suppress viral infection (22). The selective CDK9 inhibitor FIT-039 suppressed the replication of HAdV-5 genomic DNA and the transcription of the HAdV-5 early gene E1A (91). The CDK7 inhibitor LDC4297 has antiviral activity against HAdV-2 (92).

#### Targeting the late phase of infection

The inosine monophosphate dehydrogenase (IMPDH) inhibitor mycophenolic acid (MPA) and the dihydroorotate dehydrogenase (DHODH) inhibitor A3, which are involved in the *de novo* synthesis of purines and pyrimidines, respectively, led to a reduction in HAdV-5 DNA replication (93, 94).

The host factor retinoic acid receptor β (RARβ) also plays an important role in HAdV replication. The blockade of HAdV-5/7/55 replication by the RARβ agonist tazarotene occurred after early HAdV protein expression, preventing HAdV infection from progressing into the late stage (95).

Verdinexor (KPT-335) was reported to have an anti-HAdV-5 effect by targeting exportin 1 to block the nuclear export of critical viral components, thereby preventing late viral RNA from being exported to the cytoplasm for translation (86, 96).

[Co(NH_3_)_6_]Cl_3_, a transition metal complex with a high positive charge density, has demonstrated antiviral activity towards HAdV-7 (97). Mechanistically, it binds strongly to negatively charged nucleotides and then causes the condensation of viral dsDNA into a toroidal-like superstructure, disturbing the viral DNA packaging process.

HAdV-2/14 were also found to be highly sensitive to nelfinavir, the active ingredient of the HIV aspartyl protease Viracept. It has been demonstrated that nelfinavir inhibits the migration of HAdV particles without perturbing other replication steps (98).

Although further studies are needed to fully characterize the mechanisms of action of these compounds and molecules, these findings provide novel insights into the HAdV replication cycle.

## Prospects

The large increase in mortality caused by severe pneumonia associated with HAdV infection emphasizes the urgent need for effective and safe anti-HAdV therapy. Current therapeutic modalities for HAdV infection are limited, including preemptive suppression of HAdV replication using antivirals and/or immunotherapy. Immunotherapy approaches, including infusion of HAdV-specific T lymphocytes, are developed and promising, but also are very labor intensive and expensive, restricting their application. In parallel, the search for antivirals effective in treatment and prevention of diseases caused by HAdV continues. Most of the anti-HAdV compounds discussed in this review have displayed some efficacy *in vitro*, as have others *in vivo*, and some compounds are even being tested in clinical trials. HAdV exhibits species-restricted phenotypes, making studying disease progression and testing anti-HAdV agents in animal models particularly challenging. However, several existing models, such as immunosuppressed Syrian hamsters, humanized transgenic mice, and lung organoids, are promising and have the potential to evolve rapidly in the coming years (21, 37, 42, 58, 99–101). Given that some compounds have been clinically approved for other diseases, existing information on pharmacologic parameters might aid more extensive *in vivo* studies on their anti-HAdV properties. New anti-HAdV compounds and therapeutic targets could be developed further with the help of advanced drug screening platforms and methods, together with developed animal model. Thus, we are optimistic that suitable anti-HAdV agents will soon be identified and developed.
